# An Edge-Computing-Based Emotion-Aware Adaptive Lighting System for Intelligent Cockpits

**DOI:** 10.3390/s26113489

**Published:** 2026-06-01

**Authors:** Lei He, Ning Jia, Jiaqi Zhao

**Affiliations:** National Key Laboratory of Automotive Chassis Integration and Bionics, Jilin University, Changchun 130025, China; jianing24@mails.jlu.edu.cn (N.J.); jiaqiz23@mails.jlu.edu.cn (J.Z.)

**Keywords:** intelligent cockpit, driver monitoring system, emotion perception

## Abstract

As intelligent cockpits transition into the “third living space”, traditional driver monitoring systems face limitations such as rigid monitoring, computationally intensive algorithms, and insufficient engineering robustness. This paper proposes an edge-computing-based emotion-aware ambient lighting system, forming a complete loop of emotion perception–decision–adaptation. A lightweight emotion recognition network is designed for edge computing: the Mini_XCEPTION architecture is optimized with depthwise separable convolutions to reduce parameters, and a Gaussian-smoothed weighted cross-entropy loss function is used to address class imbalance and ambiguous emotion boundaries. After INT8 quantization, the model achieves 47 FPS real-time inference on a Raspberry Pi (Raspberry Pi Ltd., Cambridge, United Kingdom). A high-concurrency asynchronous software–hardware architecture based on PyQt5 5.15.6 and QThread5.15.6 is built, with a serial communication mechanism featuring fixed-length frames and fault recovery to improve the robustness of the hardware-in-the-loop system. Breaking the rigid alarm mode, an emotion–HSV lighting mapping matrix is established based on the Russell Valence-Arousal model, combined with 0.1 Hz bionic breathing rhythm for non-intrusive feedback. An FSM-controlled HSV lighting policy with 0.1 Hz breathing-light feedback was implemented on an in-cabin HIL platform. In a 12-participant simulated road-rage test, the intervention reduced FER-based anger recovery time by 42.6%; independent physiological validation remains necessary.

## 1. Introduction

Road safety remains the fundamental bottom line for the global automotive industry. As intelligent cockpits rapidly transition into the “third living space,” active safety technologies are evolving from traditional physical sensing systems, such as Autonomous Emergency Braking (AEB), to advanced observable driver-state monitoring. This evolution aligns with the emerging paradigm of Human-Centric Lighting (HCL) in automotive ambient HMI. In this framework, in-vehicle illumination can serve as a continuous, non-intrusive feedback interface [1]. With the widespread adoption of Level 2+ ADAS, monitoring drivers’ affective states during control handover has become increasingly important. High-arousal negative expressions may be associated with risk-taking behaviour, whereas low-arousal negative states may accompany reduced situational awareness [2,3].

Facial expression recognition (FER) is a core technology in affective computing for driver monitoring. Early methods relied on handcrafted features and shallow classifiers [4,5], but their robustness is limited in dynamic and unstructured vehicle-cabin environments. Deep learning methods, especially convolutional neural networks, have therefore become dominant because they can automatically learn hierarchical semantic features and improve recognition accuracy [6,7,8]. However, their generalization strongly depends on dataset quality and diversity. Laboratory-controlled datasets such as JAFFE [9] and CK+ [10] mainly contain posed expressions and lack real-world cabin interference, which may cause poor transfer to in-vehicle scenarios. To reduce this domain gap, this study adopts the uncontrolled FER2013 dataset [11]. Although noisy and low-resolution, FER2013 better reflects non-cooperative imaging conditions such as lighting variation, head movement, and partial occlusion in vehicle cabins.

Translating these highly accurate deep learning models into practical automotive applications requires deploying them on edge devices with specific hardware constraints. Standard deep networks typically rely on parameter-heavy fully connected layers, creating a severe “memory wall” and computational bottleneck that prevents real-time execution on resource-constrained platforms. To address this, the industry has turned to lightweight architectures. While mainstream lightweight networks like SqueezeNet [12] and the MobileNet series [13,14] achieve substantial parameter reduction through structural innovations like depthwise separable convolutions, Mini_XCEPTION [15] represents an extreme architectural simplification specifically tailored for real-time edge computing systems. By entirely removing fully connected layers and replacing them with global average pooling (GAP), Mini_XCEPTION massively compresses the model footprint while maintaining highly competitive accuracy on complex datasets like FER2013 [16]. Additionally, highly robust face detection and tracking solutions are essential to maintain stable facial capture across different seating postures and severe occlusions [17].

While accurate perception is the foundation, an intelligent cockpit should also provide adaptive feedback. Conventional driver monitoring systems generally rely on unidirectional alarm cues. In contrast, ambient lighting offers a continuous, non-intrusive feedback channel. Based on heuristic colour-psychology principles, this study uses dynamic lighting as an adaptive visual cue for high-arousal negative expressions [18].

Despite significant theoretical progress in both FER and human–machine interaction, translating these laboratory-level achievements into practical, production-ready cockpits faces four major engineering challenges. First, there is a persistent imbalance between perception accuracy and inference latency on edge hardware. Second, traditional facial tracking algorithms suffer from tracking failures and high false-positive rates under extreme lighting or complex occlusions [19]. Third, insufficient perceptual robustness in dynamic, unstructured environments leads to a sharp decline in recognition confidence [20]. Fourth, there is a critical lack of a complete, closed-loop emotional interaction mechanism, as most existing systems stop at mere recognition and fail to implement active, state-driven adaptive visual feedback.

To address these critical shortcomings, this paper proposes an edge-computing-based emotion-aware adaptive lighting system that establishes a complete “perception–decision–adaptation” cycle. The main research contributions are summarized as follows:

Data augmentation and preprocessing in an in-vehicle environment: we introduce an optimized pipeline tailored for vehicle cabins, utilizing geometric transformations and adaptive histogram equalization to ensure algorithmic robustness against severe dynamic lighting variations.

Improved Mini_XCEPTION network design and training: We design a lightweight architecture utilizing depthwise separable convolutions and a Gaussian-smoothed weighted cross-entropy loss function. This architecture overcomes edge device hardware constraints to achieve high-speed real-time inference (>30 FPS) on standard CPU platforms.

Highly robust face detection and adaptive ROI extraction: a hybrid detection and tracking strategy is proposed, optimizing the Region of Interest (ROI) cropping logic to guarantee stable facial capture across various complex driving postures.

FSM-Coupled Lightweight Perception and High-Concurrency Control Architecture: We embed Mini_XCEPTION into a four-stage FSM-based closed-loop pipeline, rather than treating it as an isolated classifier. Using PyQt55.15.6/QThread asynchronous multithreading, the system smooths emotion probabilities, converts them into FSM transition signals, and supports low-latency adaptive emotion-lighting control on edge devices.

## 2. Design of the Emotion Recognition Algorithms

The design of driver emotion recognition algorithms is essentially a balancing act between high-dimensional feature representation capabilities and the constraints of embedded resources. This research will detail a design process for driver emotion recognition algorithms tailored for in-vehicle embedded platforms.

### 2.1. Dataset Preparation and Preprocessing

This paper uses FER2013, released in a Kaggle competition, as the primary training dataset. It contains 35,887 grayscale facial images with a resolution of 48 × 48 pixels, covering seven basic emotions: anger, disgust, fear, happy, sadness, surprise, and neutral. Its low resolution, low signal-to-noise ratio, multi-angle faces, occlusions, and uneven lighting resemble non-cooperative in-vehicle camera conditions, thereby supporting model generalization in real-world cockpit environments.

To convert unstructured raw data into standard tensors suitable for convolutional neural networks, this paper constructs a pre-processing pipeline encompassing weighted greyscaling, bicubic interpolation scaling, histogram equalisation, and Z-score normalisation, aiming to strike a balance between in-vehicle computational constraints and image feature fidelity [21].

**Figure 1:**
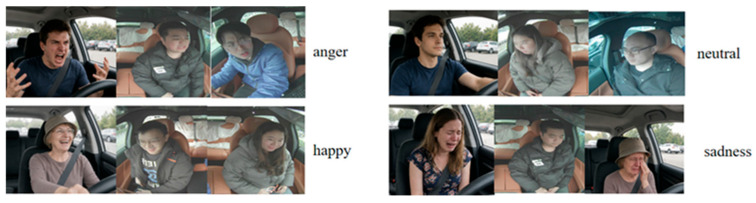
Representative samples from the independent in-cabin cross-domain test set.

Beyond training on the FER2013 dataset, we incorporate a real-world in-vehicle emotion dataset as an independent cross-domain test set to objectively assess the model’s generalization in complex vehicular environments. Figure 1 shows some sample data.

### 2.2. Mini_XCEPTION Network Architecture Design

In response to the computational and storage constraints of in-vehicle embedded environments, this research presents an improved, lightweight Mini_XCEPTION convolutional neural network.

**Figure 2:**
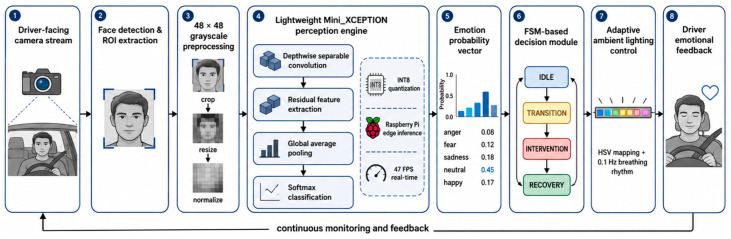
Edge-oriented lightweight perception pipeline for DMS deployment.

Figure 2 illustrates the edge-oriented DMS perception pipeline. The driver facial ROI is converted into a 48 × 48 grayscale tensor and processed by the lightweight Mini_XCEPTION engine. Using depthwise separable convolution, global average pooling, and INT8 quantization, the model enables real-time Raspberry Pi inference and outputs emotion probabilities for FSM-based adaptive lighting control.

#### 2.2.1. Architectural Design Philosophy: Deep Compression and Fully Convolutional Architecture

Mini_XCEPTION is designed to address the resource bottleneck of deep CNNs on automotive edge devices. Based on the Xception principle, it introduces lightweight structural optimization through spatial–channel decoupling, a fully convolutional architecture, and structured regularization.

Spatial–channel decoupling is achieved using depthwise separable convolutions, where 3 × 3 channel-wise convolutions extract spatial features and 1 × 1 point-wise convolutions fuse channel information. This design reduces the computational redundancy of standard convolutions.

The fully convolutional structure removes parameter-heavy fully connected layers, which often dominate model size. Combined with structured regularization, this architecture substantially compresses parameters while improving generalizable facial feature learning on limited emotion datasets.

**Figure 3:**
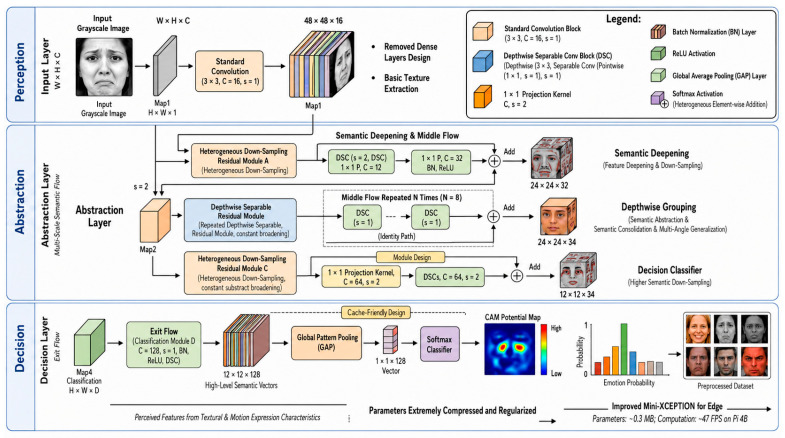
Topological architecture of the improved Mini_XCEPTION lightweight convolutional neural network.

A Modular Stream-Based Feature Pyramid is shown in Figure 3: Figure 3 illustrates the structural pruning strategy of the improved Mini_XCEPTION network. By replacing standard convolutions with depthwise separable convolutional blocks and removing fully connected layers through global average pooling, the model significantly reduces parameter redundancy and memory overhead. Residual shortcuts further preserve facial features during down-sampling, making the network suitable for continuous DMS inference on embedded edge platforms. This flow-based design sequentially extracts textures, enhances semantic features, and maps high-dimensional representations to emotion categories, thereby encoding raw pixels into compact emotional semantic vectors.

#### 2.2.2. Core Operators: Depthwise Separable Reconstruction Tailored for Edge Computational Bottlenecks

When deploying deep learning models on automotive edge devices, systems encounter severe memory bandwidth constraints and computational “memory walls”. Traditional standard convolutions inevitably incur computational redundancy across both spatial and channel dimensions during feature extraction. To address this, the proposed system employs Depthwise Separable Convolutions (DSC) to replace the most computationally intensive standard convolutional layers within the network.

Based on the premise of spatial–channel decoupling, DSC decomposes standard 3D convolutions into two independent operations: channel-wise spatial filtering and pixel-wise channel fusion. Under this architecture—predominantly utilizing 3 × 3 convolutional kernels—the computational complexity is drastically compressed to approximately one-ninth of that required by traditional convolutions. This fundamental reconstruction of the underlying operators effectively eliminates memory access latency, establishing the computational foundation necessary to achieve real-time inference at 47 FPS on a pure CPU platform.

#### 2.2.3. Structural Regularization and Nonlinear Activation Strategies

Uncontrolled automotive datasets such as FER2013 are affected by abrupt illumination changes, occlusions, and imbalanced class distributions, which increase the risk of unstable training and overfitting in deep networks. To improve feature robustness, the proposed Mini_XCEPTION model integrates Batch Normalization (BN) and ReLU activation throughout the fully convolutional feature extraction process.

BN reduces internal distribution shifts caused by dynamic in-cabin lighting, stabilizing gradient propagation and allowing more reliable optimization. ReLU introduces nonlinear sparse activation, helping the network focus on discriminative facial cues while reducing redundant feature coupling. In addition, residual connections are retained in heterogeneous down-sampling modules so that low-level perceptual information and error signals can be effectively propagated across layers, thereby maintaining training stability after the removal of fully connected layers.

#### 2.2.4. Head Design: Global Average Pooling Replaces Fully Connected Layers

**Figure 4:**
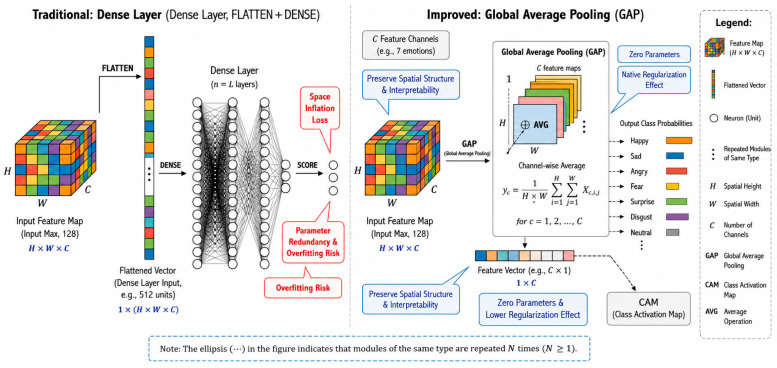
Comparison of feature dimension reduction mechanisms between traditional fully connected (dense) layers and global average pooling.

Departing from the traditional “flattening + fully connected” architecture, we adopt global average pooling (GAP) as the final classifier to address the issues of parameter redundancy and loss of spatial information in fully connected layers, as shown in Figure 4.

Traditional fully connected layers account for over 80% of the total parameters, which can easily cause memory overflow in in-vehicle devices; the flattening operation disrupts the spatial topology of feature maps, increasing the risk of overfitting.

GAP generates an output vector by calculating the average of the spatial dimensions of the feature maps. If the final convolutional layer outputs K H × W feature maps, the mathematical expression is:

(1) $$y_{k}=\frac{1}{H+W}\sum_{i=1}^{H}\sum_{j=1}^{W}x_{k}\left(i,j\right)$$

The GAP layer contains no learning parameters, enabling native regularization and improving generalization on small datasets; at the same time, it preserves spatial structure and supports the generation of activation maps (CAMs), enhancing the algorithm’s interpretability.

#### 2.2.5. Comparative Analysis of Model Complexity

To objectively validate the lightweight advantages of Mini_XCEPTION, this study selected mainstream deep convolutional networks (VGG16, ResNet50) and the lightweight benchmark MobileNetV1 as baselines and conducted theoretical and experimental comparisons across three dimensions: parameter size, computational cost, and inference latency, as shown in Table 1.

As shown in the table, Mini_XCEPTION is the only evaluated model exceeding 30 FPS on the Raspberry Pi 4B while maintaining the smallest model size and computational cost in Table 1.

**Figure 5:**
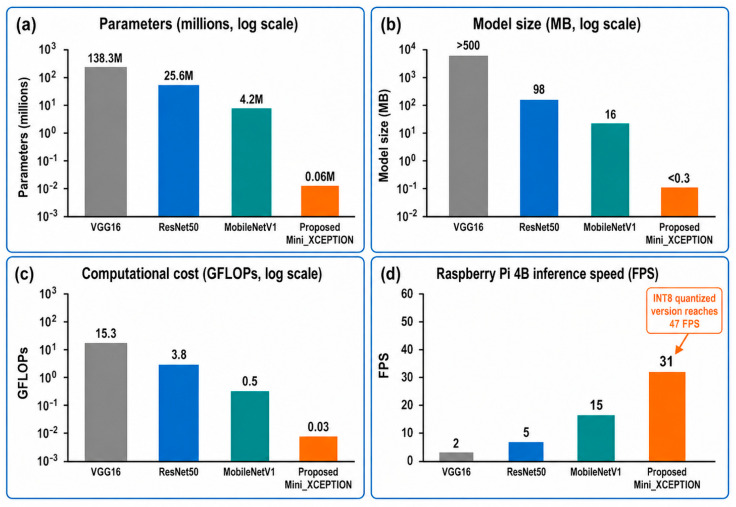
Complexity and edge-inference comparison between CNNs and Mini_XCEPTION: (**a**) number of parameters; (**b**) model size; (**c**) computational cost; and (**d**) Raspberry Pi 4B inference speed.

Figure 5 graphically compares the model complexity and edge-inference performance listed in Table 1. The proposed Mini_XCEPTION achieves much lower parameters, model size, and FLOPs than VGG16, ResNet50, and MobileNetV1, while maintaining real-time Raspberry Pi inference, confirming its suitability for resource-constrained DMS deployment.

### 2.3. Model Training and Hyperparameter Tuning

#### 2.3.1. Objective Function Design: Gaussian-Smoothed Weighted Cross-Entropy

Given the one-hot encoded label characteristics of the FER2013 multi-classification task, we selected classification cross-entropy as the base loss function. To address the issue of model bias toward the majority class caused by the dataset’s extreme long-tail distribution, we introduced a category weighting strategy to construct a weighted classification cross-entropy loss function.

First, based on the number of samples in each category in the training set, we calculate the penalty weight W_j_ for emotion category j. The calculation logic is “the fewer the samples, the greater the weight”:

(2) $$W_{j}=\frac{N_{total}}{C\cdot n_{j}}$$

Here, N_total_ represents the total number of samples in the training set and C = 7 represents the total number of classes and the number of samples in class j. Using this formula, minority emotion classes receive larger weights than majority classes.

The modified loss function $L_{weighted}$ is defined as:

(3) $$L_{weighted}=-\sum_{i=1}^{M}\sum_{j=1}^{C}W_{j}\cdot y_{i,j}\cdot log\left(\hat{y_{i,j}}\right)$$

M: current batch size.

$\hat{y_{i,j}}$ : model prediction probability.

This function increases the penalty for minority-class samples, encouraging the model to focus on difficult tail-distribution emotions. However, because weighted cross-entropy still relies on rigid one-hot labels and ignores semantic proximity among ambiguous emotions, it is used only as an intermediate objective before introducing the final GSWCE loss.

Although weighted cross-entropy improves minority-class learning, it still uses one-hot labels and ignores inter-emotion proximity. To handle ambiguous in-vehicle expressions caused by lighting changes, occlusion, and non-frontal poses, Gaussian-smoothed targets are introduced in the valence–arousal space.

(4) $${\tilde{y}}_{i,j}=\frac{exp(-\frac{{d(e_{i},e_{j})}^{2}}{{2\sigma }^{2}})}{\sum_{k=1}^{C}exp(-\frac{{d(e_{i},e_{k})}^{2}}{{2\sigma }^{2}})}$$

where $d(e_{i},e_{j})$ denotes the distance between emotion categories $e_{i}$ and $e_{j}$ in the valence–arousal space and σ controls the smoothing strength.

(5) $$L_{GSWCE}=-\frac{1}{M}\sum_{i=1}^{M}\sum_{j=1}^{C}\omega_{j}{\tilde{y}}_{i,j}log(p_{i,j})$$

Therefore, the final deployed Mini_XCEPTION model is trained with the proposed GSWCE, which combines class-frequency weighting with Gaussian emotion-space smoothing. This design addresses long-tail class imbalance, reduces over-confident learning from ambiguous labels, and improves sensitivity to high-risk negative emotions such as anger, fear, and disgust.

#### 2.3.2. Optimizer Selection: Adaptive Moment Estimation

The Adam (Adaptive Moment Estimation) optimizer was selected to perform gradient descent optimization. This optimizer combines the core strengths of momentum and RMS Prop, allowing for adaptive adjustment of learning rates: The momentum mechanism maintains the inertia of the update direction through first-order gradient moment estimation, accelerating convergence and preventing the algorithm from getting stuck in local minima. The adaptive feature dynamically adjusts the step size based on the second-order gradient moment estimates, allocating larger step sizes to sparse features corresponding to minority classes. The specific parameter settings are initial learning rate α = 0.001, first-order moment decay rate β_1_ = 0.9, and second-order moment decay rate β_2_ = 0.999.

#### 2.3.3. Regularization and Strategies to Prevent Overfitting

To further enhance Mini_XCEPTION’s generalization ability in uncontrolled in-vehicle environments, this study introduces the following mechanisms in addition to data augmentation:

Dropout regularization: A dropout layer is introduced after the convolutional layers (with a dropout rate set to 0.25–0.5). As noted by Srivastava et al., dropout prevents complex co-adaptation among neurons by randomly “disconnecting” neural connections during training, thereby effectively suppressing overfitting.

Early stopping mechanism: Monitor the validation set loss. When the loss does not show a significant decrease (Δ_min_ < 0.001) over 10 consecutive epochs, training is forcibly terminated to prevent the model from overfitting to the training set.

#### 2.3.4. Summary of Experimental Conditions and Parameters

All experiments were conducted using the TensorFlow 2.6.0 deep learning framework. The specific software and hardware environments, as well as the hyperparameter configurations, are shown in Table 2.

As the final training objective of the model, GSWCE enables robust classification under ambiguous in-vehicle emotion distributions.

#### 2.3.5. Model Evaluation Results

**Figure 6:**
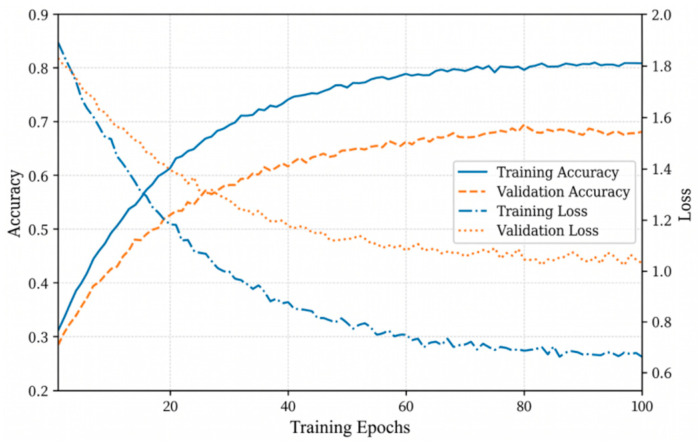
Convergence curves for loss and accuracy during the training of the GSWCE-based Mini_XCEPTION model.

To visually assess model performance, this paper uses Keras’s History object to record the training set loss, validation set loss, training set accuracy, and validation set accuracy for each epoch; the result is shown in Figure 6.

The plotted training curves clearly show the following: The loss decreases rapidly during the first 15 epochs without obvious overfitting, indicating the effectiveness of dropout and data augmentation. Validation accuracy peaks around epoch 40, after which the ReduceLROnPlateau callback reduces the learning rate and further stabilizes model convergence.

**Figure 7:**
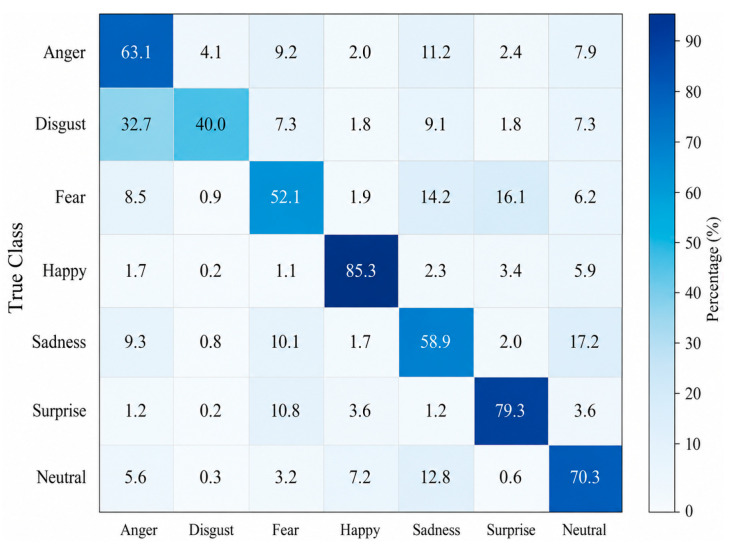
Normalized confusion matrix of the improved Mini_XCEPTION model.

To evaluate perception performance beyond overall accuracy, validation-set analysis was conducted using class-level metrics. Because FER2013 is imbalanced, Figure 7 presents the normalized confusion matrix and Table 3 reports the precision, recall, and F1-score for each emotion category.

To evaluate the effect of quantization, the FP32 and INT8 Mini_XCEPTION models were compared on the Raspberry Pi 4B in terms of model size, inference latency, FPS, and accuracy change, as reported in Table 4.

The experimental data shows the following: After quantization, the model size was reduced to 68 KB, substantially lowering memory-access latency and bandwidth overhead. On the Raspberry Pi platform, inference speed increased from 31 FPS to 47 FPS, leaving computational margin for face detection and UI rendering. After representative-data calibration, INT8 quantization caused only a 0.8% accuracy loss, preserving model precision.

To evaluate both class weighting and robust loss design, CE, WCE, Focal Loss, Class-Balanced Focal Loss, and the proposed GSWCE were compared under identical training settings. CE served as the unweighted baseline, WCE isolated the effect of class weighting, and GSWCE further introduced Gaussian emotion-space smoothing. The evaluation focused on accuracy, macro-F1, and the recall of high-risk or minority emotions, including anger, fear, and disgust.

As shown in Table 5, WCE improves the recall of anger, fear, and disgust from 51%, 45%, and 42% to 62%, 55%, and 58%, respectively. GSWCE further increases these values to 66%, 59%, and 62%, without additional inference-time cost on the Raspberry Pi. GSWCE is selected as the final loss function for the deployed Mini_XCEPTION model.

High accuracy of deep learning models on controlled datasets often degrades in real-world scenarios. To verify the engineering robustness of the proposed improved Mini_XCEPTION and image preprocessing pipeline, the model was deployed on the in-cabin dataset for cross-domain testing without fine-tuning, as shown in Table 6.

Although cross-dataset facial differences reduce overall accuracy by 9.3% relative to FER2013, our GSWCE strategy maintains a practical 54.2% recall for high-risk emotions like anger. This confirms the algorithm’s robustness for non-cooperative in-cabin environments.

This research presents a lightweight and robust driver emotion recognition algorithm for in-vehicle embedded deployment. To address the uncontrolled characteristics and long-tail distribution of FER2013, we constructed a preprocessing pipeline with histogram equalization and geometric transformations and trained the model using Gaussian-smoothed weighted cross-entropy. An improved Mini_XCEPTION network was developed using depthwise separable convolutions, residual connections, and global average pooling, substantially reducing model parameters and computational cost for edge devices. Through post-training quantization, the FP32 model was converted into INT8 TFLite2.6.0 format, reducing the model size to 68 KB while maintaining an accuracy loss below 1%. The final model achieved 47 FPS on the Raspberry Pi platform, meeting the real-time requirements of in-vehicle deployment.

## 3. System Software Design

The previous section developed an improved Mini_XCEPTION model to address limited in-vehicle computing resources and long-tail emotion distributions. However, high-performance recognition algorithms must be transformed into deployable engineering systems for practical use. In driver emotion recognition, the key challenge has shifted from accuracy improvement alone to real-time deployment under resource-constrained conditions, because algorithmic advantages do not necessarily ensure engineering feasibility [22].

Therefore, this section presents the software implementation of the driver emotion monitoring system. Since unoptimized deep models often suffer from high inference latency on embedded platforms and cannot meet the real-time requirements of driving scenarios [23], the system follows a lightweight, modular, and high-concurrency design. Implemented in Python 3.8 with PyQt55.15.6, OpenCV4.5.3, and TFLite2.6.0, it focuses on asynchronous decoupling of video capture, inference, and GUI refresh, emotion-output jitter suppression, and scalable modular architecture [24].

### 3.1. Software Architecture and Data Flow

To reduce coupling between functional modules and enhance the system’s maintainability and scalability, this system has moved away from the traditional monolithic script structure and adopted a modular, layered architecture.

#### 3.1.1. Layered Design Based on the MVC Pattern

**Figure 8:**
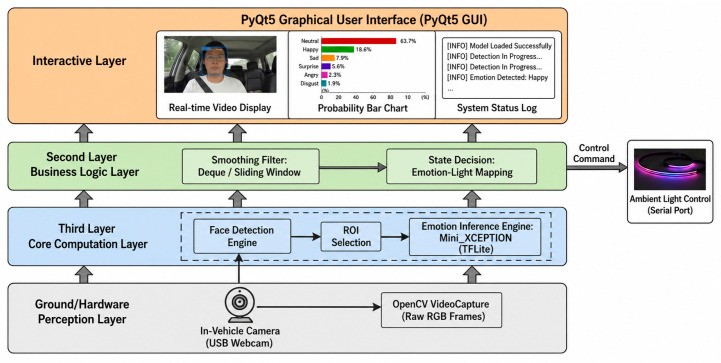
System-level software architecture diagram based on the MVC pattern.

The system adopts the MVC design pattern to separate business logic, data processing, and user interaction. This layered design reduces code complexity [25] and improves the portability of algorithm modules across hardware platforms. As shown in Figure 8, the software architecture consists of four logical layers.

The hardware perception layer acquires RGB video streams from the in-vehicle camera through OpenCV. The core computation layer performs face detection and emotion inference in parallel. The business logic layer applies moving-average smoothing and generates lighting commands based on the dominant emotion. Finally, the PyQt5-based interaction and display layer updates the video stream, emotion probability bars, and system logs through the signal-slot mechanism.

#### 3.1.2. Data Flow and Inter-Module Interface Design

Data flows within the system follow a unidirectional closed-loop principle to ensure data traceability:

- Input: the camera captures raw frames $I_{raw}$ ;
- Process: I_raw_ → Face Detection → Excerpt I_face_ → Preprocessing → $\mathrm{tensor}\;T_{input}$
- Inference: $T_{input}\to CNN\;\mathrm{Model}\to \mathrm{Probability}\;\mathrm{vector}$ ;
- Logic: P→Smoothing algorithm→Stable L ;
- Output: L → Trigger a GUI update and send a command via the serial port.

### 3.2. Implementation of Key Functional Modules

The core challenge of this system lies in how to simultaneously perform four high-frequency tasks—video stream capture, face detection, deep model inference, and UI refresh—while operating within limited CPU resources. To ensure the system’s stability and real-time performance during prolonged operation, this section will provide a detailed explanation of the code logic and optimization strategies for the key modules.

#### 3.2.1. Asynchronous Video Capture and UI Update

To thoroughly resolve the issue of UI blocking caused by Python’s global interpreter lock, this system has built an asynchronous video capture engine based on the QThread class in PyQt5. In addition to basic frame reading functionality, it also integrates real-time FPS calculation and an automatic resource release mechanism.

**Figure 9:**
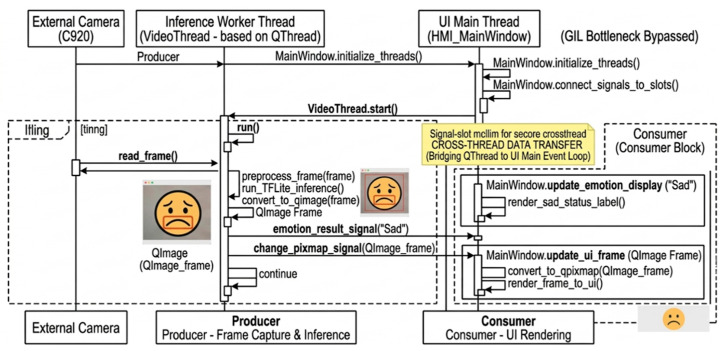
Timing diagram of a multithreaded asynchronous pipeline based on the QThread signal-slot mechanism.

Signal-and-Slot Architecture Design Data communication between the subthreads and the main UI thread employs the “signal-and-slot” mechanism, eliminating potential race conditions caused by shared memory in multithreading, as shown in Figure 9, the highlighted yellow block indicates the PyQt5 signal-slot mechanism used for thread-safe cross-thread data transfer. The inference worker thread transmits the emotion-recognition result and processed image frame to the UI main thread through queued signals, enabling asynchronous interface updates without directly operating on UI elements from the worker thread.

#### 3.2.2. Face Detection and Adaptive ROI Extraction

To ensure a system frame rate of over 30 FPS in an embedded CPU environment, this system employs a Haar cascade classifier—which has lower computational complexity—to perform initial face screening.

The key parameters of detect Multi Scale were tuned to balance detection accuracy and false-positive suppression. Specifically, ‘scaleFactor’ was set to 1.1 to detect faces at multiple scales while limiting processing time, and minNeighbors was set to 5 to filter background regions resembling faces. After obtaining the face coordinates (x, y, w, h), a standardized ROI extraction procedure was applied. The detected face region was converted to grayscale, resized to 48 × 48 pixels using bicubic interpolation, and normalized to [0, 1] for Mini_XCEPTION inference.

#### 3.2.3. Smoothing of Emotion Recognition Results

**Figure 10:**
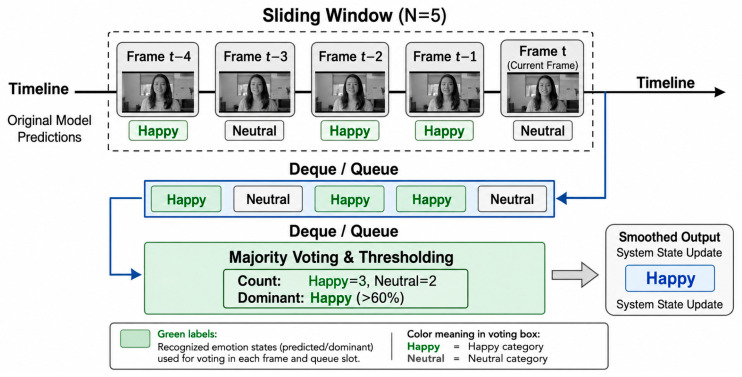
Schematic diagram of a sliding-window-based majority voting anti-jitter algorithm.

Single-frame emotion predictions are sensitive to illumination changes and facial micro-expression transitions, which may cause abrupt label fluctuations and frequent ambient-light switching. To improve temporal stability, the system adopts a sliding-window majority voting strategy based on the continuity of emotional states, as shown in Figure 10.

During algorithm implementation, the queue is first initialized by creating a fixed-length deque: ‘history_queue = deque(maxle*N* = 5)’. After each model inference, the label with the highest predicted probability for the current frame is enqueued; if the queue is full, the oldest label is automatically dequeued. Based on the voting decision formula:

(6) $$L_{final}=argmax(Count\left(c\in history_{queue}\right))$$

Determine the label with the highest frequency in the statistical queue as the final output.

This configuration reflects a trade-off between latency and robustness. Preliminary tests showed that small windows respond quickly but increase false triggers, whereas larger windows suppress noise but introduce excessive latency. Therefore, *N* = 5 and θ = 0.6 were selected, reducing 1–2 frame jitters while keeping end-to-end latency within 300 ms, as shown in Table 7.

Based on the latency–jitter trade-off in Table 7, *N* = 5 and θ = 0.6 were selected, limiting short-term label fluctuations while keeping end-to-end latency within approximately 300 ms.

**Figure 11:**
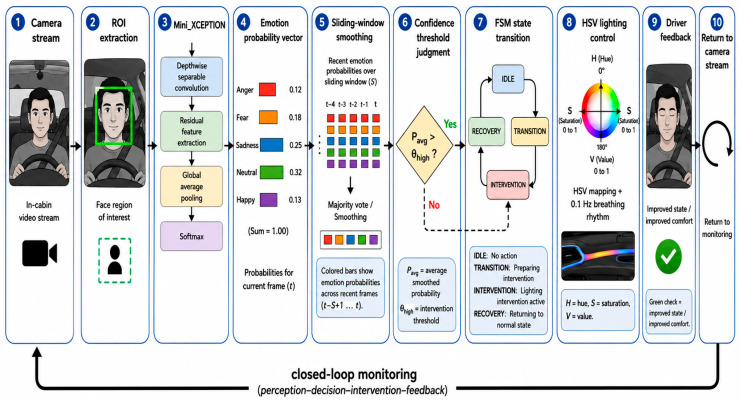
Synergistic coupling between the lightweight perception engine and FSM-based adaptive decision logic.

Figure 11 illustrates how the lightweight Mini_XCEPTION engine is coupled with the FSM-based decision logic. The emotion probability vector is temporally smoothed and converted into FSM transition conditions, enabling the system to switch among monitoring, transition, intervention, and recovery states. This design transforms the network from an isolated classifier into a real-time control signal generator for closed-loop DMS intervention.

## 4. Emotion-Aware Adaptive Lighting Strategies

As discussed earlier, the proposed Mini_XCEPTION-based model and PyQt5 multithreading architecture enable lightweight edge perception and stable system implementation [26,27,28]. However, in the driver–vehicle–road closed loop, perception is only the starting point. The key to transforming the smart cockpit from a passive tool into an active partner lies in making ergonomic decisions and interventions based on the driver’s psychological state.

### 4.1. Design Approach of Adaptive Ambient Lighting Feedback

Traditional driver monitoring systems usually follow a unidirectional “trigger-alarm” logic, which provides limited opportunities for feedback adjustment. Next-generation smart cockpits therefore require a closed-loop emotion-aware feedback system [29].

Recent studies show that adaptive illumination, including dynamic correlated colour temperature (CCT) and illuminance, can modulate driver alertness [30], while human-centric designs emphasize synchrony between cabin environments and implicit driver states [31]. Based on these principles, this study develops a deployable, low-latency “perception–intervention–feedback evaluation–readjustment” cycle that adaptively regulates lighting until the driver’s emotional state returns to a safe level.

Due to its round-the-clock coverage and contactless nature, the lighting environment is the preferred medium for in-vehicle emotion-aware feedback. Drawing inspiration from non-visual biological effects, the system utilizes ambient lighting as an adaptive visual feedback mechanism. By dynamically adjusting the hue, saturation, and brightness, the system aims to provide heuristic environmental cues that guide the driver toward a more stable visual-feedback condition.

Based on the above theory, this research proposes a “colour-compensatory lighting intervention strategy,” the core of which is to utilize the perceived properties of lighting to provide adaptive visual cues for detected negative-expression states.

### 4.2. An “Emotion-Lighting” Mapping Model Based on a Colour-Compensatory Strategy

To implement adaptive lighting control, an initial emotion–lighting mapping matrix was designed using Russell’s valence–arousal framework and prior lighting-design principles. The matrix defines candidate HSV settings for each model output and serves as a heuristic control policy in the present prototype.

#### 4.2.1. Closed-Loop Control Logic: Dynamic Intervention Model Based on Finite State Machine

To ensure the continuity and responsiveness of adaptive lighting feedback, this system designs a four-stage finite state machine (FSM) as the decision-making core [32,33,34]. By performing time-domain integration on the real-time emotion confidence vector, the model achieves a seamless transition from static recognition to a closed-loop dynamic feedback system.

IIdle State: The system continuously monitors driver facial features at a frequency of 47 FPS. When the average confidence of the target negative emotion within the sliding window, Pavg, remains below the preset threshold\ $\theta_{triggere}$ (0.75), the system maintains the baseline lighting state.

Transition State: When the emotion-confidence statistics in the deque meet the trigger conditions—specifically, when the confidence exceeds the threshold for five consecutive frames—the FSM activates the transition procedure. Utilizing a linear interpolation algorithm, the system completes a smooth switch from the current colour gamut to the target lighting gamut within a 200 ms time window. This approach eliminates the visual startle response typically caused by step changes in light intensity.

Intervention State: In this state, the system executes Pulse Width Modulation (PWM) control based on a 0.1 Hz biomimetic breathing rhythm. Through the sinusoidal brightness control function B(t), the lighting intensity is dynamically adjusted to provide a continuous visual pacing cue. This biomimetic rhythm acts as an adaptive response.

Recovery State: This stage is critical to the closed-loop logic. Instead of executing an intervention of fixed duration, the system evaluates the intervention gain in real-time. The FSM determines the intervention to be effective and shifts to the recovery state only when the driver’s anger confidence stably drops below the safety threshold\ $\theta_{safe}$ (0.3) within the observation window. Subsequently, the lighting intensity is gradually faded.

This state-transition-based control logic ensures that the system can dynamically adjust intervention strategies based on the driver’s real-time feedback, achieving a closed-loop upgrade from traditional “trigger-alarm” mechanisms to a “perception-iteration” paradigm.

#### 4.2.2. Design of an Intervention Matrix for Seven Types of Emotions

Previous research studies have empirically linked anger to traffic violations [35], quantified the probabilistic impacts of emotional valence and arousal on driving tasks [36], and established angry expressions as a psychological warning indicator for aggressive driving [37]. Recognizing that facial expressions act as probabilistic indicators of internal emotional states capable of predicting potential driving behaviours via specific conditional probabilities, this paper constructs an emotion-light mapping framework to guide ambient light regulation. By incorporating the seven basic emotions from the FER2013 dataset, we designed lighting intervention strategies and organized them into a systematic lighting intervention matrix. The lighting control strategies corresponding to each emotion are detailed in the table below (Table 8).

### 4.3. Dynamic Breathing Light Effects and Sequencing Control Logic

Based on the static mapping relationships established above, this research further explores dynamic control strategies for lighting effects over time. To avoid visual interference caused by mechanical flickering, this system incorporates biomimetic breathing rhythms and time-smoothing interpolation algorithms to establish a time-sequence control logic that complies with ergonomic principles.

#### 4.3.1. A Parametric Definition of Colour Spaces

For high-arousal negative emotions such as “anger” and “fear”, while simple static cool light can have a calming effect, it lacks the ability to actively guide physiological rhythms. Breathing-style lighting can significantly reduce users’ heart rate and electrodermal activity (EDA) at the subconscious level through visual cues, with effects superior to those of static lighting.

A slow breathing frequency of 0.1 Hz enhances the asymmetry of prefrontal alpha waves, effectively buffering anxiety induced by visual stimuli [38]. Based on this, the system constructs a sinusoidal PWM brightness control function B(t). It is explicitly noted that while the system regulates lighting via HSV mapping, objective physical measurements of ambient lighting quality—such as lux, luminance, colour temperature, and flicker characteristics—were not performed with specialized optical hardware, as these photometric validations fall outside the current research scope. To ensure visual comfort, the underlying PWM frequency is set above 1000 Hz, eliminating stroboscopic flicker. While absolute photometric metrics remain to be precisely quantified in future work, this setup heuristically optimizes visual safety.

The control model is defined as follows:

(7) $$B(t)=B_{base}+A{\left(\frac{1+sin\left(\omega t+\varphi \right)}{2}\right)}^{\gamma }$$

B(t): target brightness value at time t (Duty Cycle, 0–255).

$B_{\mathrm{base}}$ : base ambient brightness, preventing visual discontinuity caused by the light turning off completely (set to 40).

A: breathing amplitude, which determines the dynamic range of the light-dark variation.

ω : angular frequency, set to ω = 2π × 0.1, corresponding to the configured breathing-light frequency of 0.1 Hz.

γ : waveform sharpness factor (Shape Factor). In this study, γ = 2.0 is used to make the peaks of the sine wave rounder, simulating the relaxed breathing pattern of “deep inhalation and slow exhalation” in humans.

When the system detects signs of road rage, rather than simply illuminating a blue light, it activates the ambient lighting to gently pulse on and off in 10-s cycles, subconsciously guiding the driver to slow their breathing.

#### 4.3.2. Linear Interpolation Smoothing Algorithm for State Transitions

As a driver’s facial expressions change continuously, it is not possible to switch the light colour based solely on the recognition results from a single frame. Sudden step changes in lighting can trigger transient glare. To ensure transition smoothness and visual comfort, this system employs a time-based linear interpolation algorithm [38]. Let the colour vector at time t_0_ be $C_{start}(H_{0},S_{0},V_{0})$ , and the target emotional color vector at time t_1_ be $C_{start}(H_{0},S_{0},V_{0})$ . The system sets the transition time window $T_{trans}$ to 200 ms. At any time t during the transition, the real-time output color $C_{out}(t)$ is calculated as follows:

(8) $$C_{out}\left(t\right)=C_{start}+(C_{target}-C_{start})\cdot \alpha (t)$$

The system sets the transition time window Ttrans to 200 ms. This duration heuristically exceeds human pupillary response latency to prevent visual startle, though rigorous absolute illuminance validation is reserved for future research, where the interpolation coefficient α(t) is:

(9) $$\alpha \left(t\right)=clamp(\frac{t-t_{0}}{T_{trans}},0,1)$$

#### 4.3.3. Implementation of Finite State Machine Control Logic

**Figure 12:**
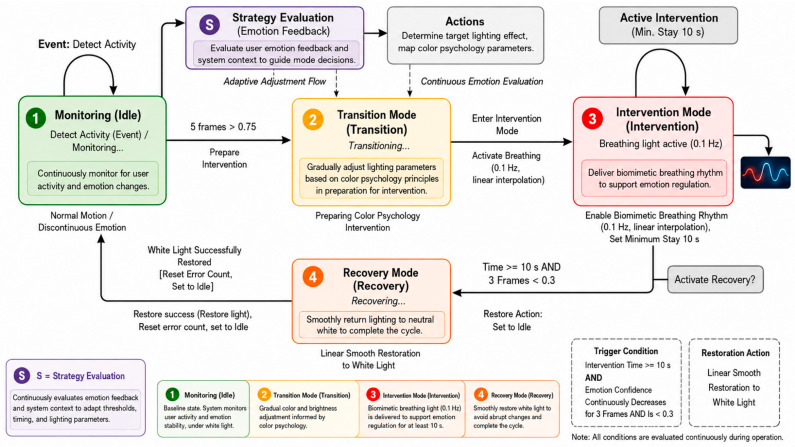
Flowchart of in-vehicle lighting sequence control based on a finite state machine (FSM).

To translate the aforementioned mathematical model into reliable software logic and fully implement the concept of “closed-loop control”, this research designed a finite state machine comprising four core states and featuring an internal feedback control loop, as shown in Figure 12.

In the monitoring state, the system maintains a comfortable baseline ambient light while continuously evaluating emotion confidence at high frequency. When the confidence of a target negative emotion exceeds the danger threshold for five consecutive frames, the FSM enters the transition state, locks the corresponding intervention colour, and applies linear interpolation to avoid abrupt visual changes. During the intervention state, the breathing-light function B(t) modulates brightness to provide continuous visual pacing. The system then evaluates intervention effectiveness in real time. If anger confidence decreases and remains below the predefined recovery threshold, the FSM enters the recovery state; otherwise, if the confidence remains high after 10 s, an adaptive reinforcement strategy is triggered. In the recovery state, inverse interpolation gradually restores the baseline lighting until emotional inertia subsides.

### 4.4. System Integration and Development of a Real-Vehicle Test Platform

**Figure 13:**
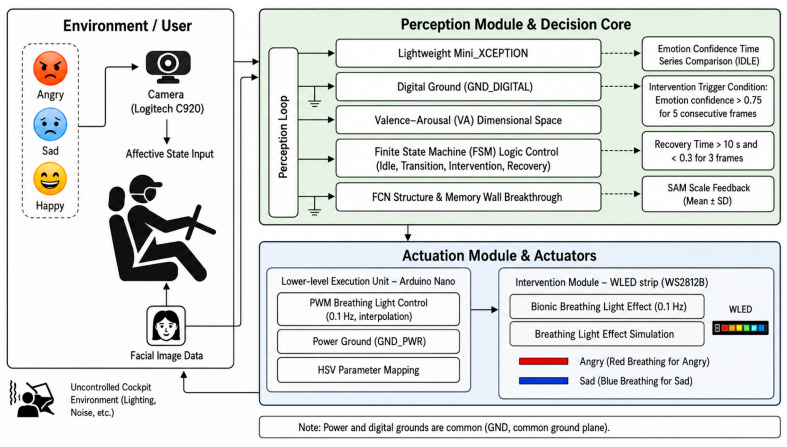
Topology and electrical isolation architecture diagram of the emotional closed-loop hardware-in-the-loop (HIL) test platform based on the EQM5 vehicle cockpit.

To validate the proposed compensatory lighting intervention strategy under realistic cockpit conditions, a complete “perception–decision–execution” hardware-in-the-loop platform was deployed in a Hongqi EQM5 cockpit, as shown in Figure 13. The platform captures real-time driver facial images, estimates emotional states, and controls the interior ambient lighting in a closed-loop manner.

The system adopts a master–slave architecture to decouple high-performance perception from low-latency execution. A Raspberry Pi 4B with 4 GB RAM serves as the perception and decision-making host, running the Mini_XCEPTION model and PyQt5 backend. Facial images are acquired using a dashboard-mounted Logitech C920 Pro RGB(Logitech Europe S.A., Lausanne, Switzerland) camera. To preserve skin-tone information under low-light conditions, the system uses adaptive illumination compensation instead of infrared imaging. A laptop connected through the in-vehicle LAN provides real-time parameter tuning and data logging.

At the execution layer, an Arduino Nano (Arduino S.r.l., Monza, Italy) generates PWM signals for the LED driver and receives commands from the Raspberry Pi through a USB-to-TTL (Future Technology Devices International Limited, Glasgow, United Kingdom) serial connection at 115,200 bps. The WS2812B full-colour LED strips (WORLDSEMI CO., LIMITED, Dongguan, China) are powered by an independent 5 V/10 A supply (MEAN WELL Enterprises Co., Ltd., New Taipei City, Taiwan), with electrical isolation and series resistors used to reduce signal ringing, voltage drops, and interference with the original vehicle electrical system.

The core inference software environment of the system is built on the Raspberry Pi operating system and receives remote commands via a Python script running on a laptop (Lenovo Group Limited, Beijing, China).

To induce authentic and varied driving emotions while ensuring safety, this paper utilises the Hongqi EQM5 cockpit (FAW Hongqi, Changchun, China) to construct an immersive multimodal stimulation system combining ‘static scenes and dynamic visuals’ and integrates a simulated driving system capable of delivering multimodal stimuli.

The experiment utilised a display screen behind the steering wheel to play high-definition first-person view traffic footage, with two emotion-inducing scenarios designed:

Scenario A: Simulated traffic congestion accompanied by frequent honking, designed to induce feelings of anger and anxiety;

Scenario B: Simulated monotonous motorway cruising accompanied by continuous white noise, designed to induce feelings of fatigue and drowsiness.

### 4.5. Analysis of Intervention Effects in Typical Scenarios

This section evaluates the proposed system using high-risk road-rage scenarios on the Hongqi EQM5 in-vehicle HIL platform. Twelve licensed volunteers participated in a within-subjects experiment comparing conditions with and without lighting intervention. To reduce learning and fatigue effects, a Latin-square counterbalanced sequence was adopted: one group first completed the no-intervention condition followed by the lighting-intervention condition, while the other group followed the reverse order. A 15 min natural-light rest interval was provided between conditions to restore emotional and visual states to baseline. The evaluation focused on emotional recovery speed and subjective psychological experience.

#### 4.5.1. Temporal Analysis of Emotional Responses in Road Rage Situations

**Figure 14:**
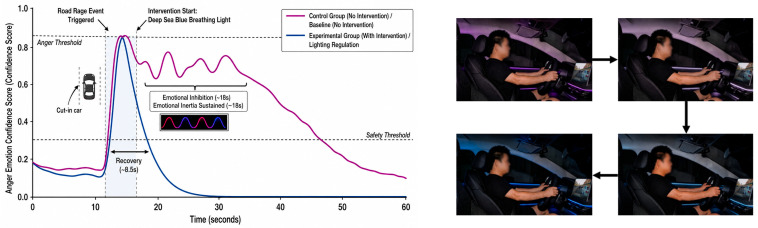
Representative anger-confidence trajectories in the road-rage scenario and the corresponding cockpit-light transition from purple to blue.

In this experiment, a malicious cut-in event was triggered at t = 10 s during driving-video playback, accompanied by a sharp horn sound through the vehicle audio system to induce anger. The system backend recorded the real-time anger confidence output by the Mini_XCEPTION model. Figure 14 compares the anger-confidence trajectories of a representative participant under the control and lighting-intervention conditions, while group-level recovery statistics are reported in Table 9. To protect participant privacy, the driver’s face was blurred. The observed facial-expression changes were consistent with those described in the manuscript. Purple lighting represents the default condition. After activation of lighting control, the driving video was paused to minimize additional external influences on the driver’s response and to isolate the effect of the lighting feedback.

Clear differences can be observed between the two response curves. Between 10 and 15 s after the cut-in event, anger confidence exceeded 0.85 in both conditions, indicating a marked anger-related model response to the stimulus. After the 0.1 Hz breathing-light feedback was activated, the duration of the peak response was noticeably shortened. In the control condition, anger confidence remained above 0.6 for approximately 18 s before gradually declining. In contrast, the intervention condition returned below the predefined threshold of 0.3 within 8.5 s.

To quantify the group-level difference, a paired-samples *t*-test was conducted on FER-based anger recovery time across the 12 participants. Lighting intervention significantly reduced recovery time relative to the control condition (17.1 ± 2.8 s vs. 9.8 ± 1.5 s), corresponding to a reduction of 42.6% (t (11) = 8.65, *p* < 0.001; 95% CI of the mean difference: 5.4–9.2 s; Cohen’s d = 3.2; Table 9). Because both intervention triggering and recovery evaluation were based on FER outputs, these results support improved system-level response efficiency rather than independently verified emotion regulation.

#### 4.5.2. Temporal Analysis of Emotional Responses in “Fatigue/Monotony” Scenarios

To evaluate the system’s response to low-arousal states, Scenario B simulated monotonous nighttime highway cruising using low-frequency tire noise and dimly lit road footage to induce fatigue-like drowsiness. Because FER2013 does not include explicit fatigue labels, this research used a preliminary heuristic proxy by mapping low-arousal states to high-confidence “sadness” classification, based on shared facial cues such as muscle relaxation and drooping eyelids.

**Figure 15:**
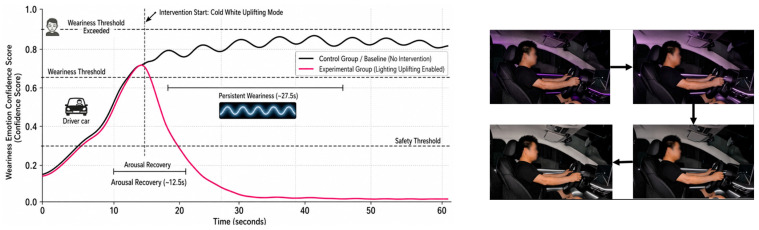
Representative trajectories of the sadness-based low-arousal proxy in the monotony scenario and the corresponding cockpit-light transition from purple to white.

A 60 s data window was recorded around the point at which each participant reached the proxy fatigue threshold. Figure 15 presents the temporal comparison after the proxy fatigue confidence exceeded the danger threshold at t = 15 s.

The temporal curve demonstrates the alerting efficacy and arousal-inducing potential of the lighting intervention mechanism:

In the control condition, proxy fatigue confidence remained high after exceeding the 0.7 threshold, fluctuating between 0.75 and 0.85. This sustained high-level response indicated continued cognitive disengagement and reduced visual attention.

In the intervention condition, once the threshold was triggered at t = 15 s, the system activated the “Cold White” mode, characterized by configured cold-white lighting feedback according to the heuristic intervention matrix. Under this alerting light stimulation, the proxy fatigue confidence showed a clear downward trend and returned to the predefined safe range more rapidly than in the control condition.

Statistical analysis further confirmed the difference in arousal recovery time between the two conditions. The paired-samples *t*-test showed a significant reduction in recovery time under lighting intervention, t (11) = 11.2, *p* < 0.001, with a 95% confidence interval of [12.0, 18.0]. The large effect size suggests that high-illuminance cold white light can provide an effective alerting cue in this preliminary small-cohort experiment, as summarized in Table 9.

This research provides a thorough a priori justification for the sample size of 12 participants from both engineering and statistical perspectives: First, the core objective of this study is to validate the engineering feasibility of an in-vehicle closed-loop feedback system; in the field of human-computer interaction, a sample of 10–15 typical users has been shown to reveal over 80% of core interaction effects. Second, although the sample size is limited, paired *t*-tests indicate that changes in both objective and subjective metrics following the intervention reached a highly significant level, with an effect size far exceeding 0.8, indicating a large effect. This statistically demonstrates that experimental conclusions retain high confidence and robustness even with a small sample size, confirming the bidirectional effectiveness of the feedback compensation mechanism. By integrating objective time-series data with subjective SAM evaluations, this experiment validated the effectiveness of the “perception–decision–intervention” closed-loop system.

## 5. Discussion

Addressing the psychological safety needs of drivers amid the transformation of the smart cockpit into a “third living space,” and addressing the issues of rigid monitoring and lack of subjective intervention in existing DMS systems, this paper proposes an emotion-responsive adaptive lighting system based on “perception–decision–adaptation” using in-vehicle edge computing. We developed a lightweight emotion perception algorithm optimized for edge computing. By employing image preprocessing, an enhanced Mini_XCEPTION network, and Gaussian-smoothed weighted cross-entropy loss, we improved the recognition rate of negative emotions. Through INT8 quantization, we achieved ultra-real-time inference on a Raspberry Pi; we developed a highly decoupled hardware-software co-design architecture and employed asynchronous scheduling and fault-tolerant communication mechanisms to enhance the robustness of the hardware-in-the-loop system. We proposed a closed-loop lighting intervention strategy that integrates colour psychology with biomimetic rhythms; stationary in-cabin simulated tests showed that the lighting-feedback condition reduced FER-based anger recovery time relative to the control.

This innovation employs a lightweight emotion recognition solution that breaks free from the path dependence of large models. By introducing GSWCE, the model addresses both class imbalance and ambiguous emotion boundaries, further improving the recall of anger, fear, and disgust without changing the network topology or increasing Raspberry Pi inference cost. By employing adaptive ambient lighting feedback and incorporating photobiological effects into driving control, the system achieves heuristic visual feedback through a 0.1 Hz biomimetic breathing rhythm, marking a transition from passive alerts to adaptive cockpit feedback. The collaborative architecture of in-vehicle hardware and software incorporates anomaly circuit-breaking and self-healing mechanisms, establishing an industrial-grade hardware-in-the-loop architecture with reference value for mass production in original equipment manufacturing (OEM) applications. However, a notable limitation is the self-referential evaluation: using the same FER model to both trigger and assess the adaptive response introduces circular logic. Independent subjective, behavioural, and physiological measures are needed to determine whether internal arousal changes beyond FER-observed facial responses. The primary contribution of this research lies in establishing an industrial-grade, low-latency edge AI deployment architecture that overcomes the computational bottlenecks of traditional DMS, utilizing heuristic lighting mapping as a proof-of-concept application.

Future research will advance the system in three key areas: first, breaking the self-referential evaluation loop by integrating independent multi-source sensor data—such as voice, steering wheel pressure, and physiological biomarkers (e.g., HRV, EDA)—to build a multimodal affective computing framework that overcomes the limitations of single-vision perception; second, introducing deep reinforcement learning algorithms to enable adaptive emotional intervention tailored to individual drivers through edge-side learning; third, advancing from stationary simulator validation to naturalistic on-road testing to assess the system’s robustness and visual ergonomic safety under dynamic real-world driving conditions. Furthermore, RGB-based facial tracking severely degrades in low-light cabin conditions. Future work will address this by integrating advanced image enhancement algorithms, such as the Low-light Unified Multi-stage Enhancement Network (LUMEN), to improve Retina Face-based detection robustness [39].

## Figures and Tables

**Table 1:** Comparison of complexity and frame rates between common convolutional neural networks and Mini_XCEPTION.

Model Architecture	Core Mechanism	Number of Parameters	Model Volume	Floating-Point Arithmetic	Raspberry Pi 4B Inference Frame Rate
VGG16	Deep Stacking	≈138.3 M	>500 MB	≈15.3 GFLOPs	<2 FPS
ResNet50	Residual Block	≈25.6 M	≈98 MB	≈3.8 GFLOPs	≈5 FPS
MobileNetV1	Depthwise Sep	≈4.2 M	≈16 MB	≈0.5 GFLOPs	≈15 FPS
Mini_XCEPTION	No fully connected layers + separable depth	≈0.06 M	>0.3 MB	≈0.03 GFLOPs	>30 FPS

**Table 2:** Summary of experimental hardware environment and deep learning model hyperparameter configurations.

Category	Project	Configuration Parameters
Hardware Environment	GPU	NVIDIA GeForce RTX4060/Laptop (8 GB VRAM NVIDIA Corporation, Santa Clara, CA, USA))
	CPU	Intel Core i9-13900HX@2.20 GHz (Intel Corporation, Santa Clara, CA, USA)
Software Environment	Framework	TensorFlow 2.6.0 + Keras 2.6.0
	Image Library	OpenCV 4.5.3
Hyperparameters	Batch Size	32
	Epochs	100
	Baseline Losses for Ablation	CE, WCE, Focal Loss, Class-Balanced Focal Loss
	Final Loss Function	Gaussian-Smoothed Weighted Cross-Entropy (GSWCE)
	Class Weights	Computed via sklearn.utils. class_weight

**Table 3:** Per-class performance of the GSWCE-trained Mini_XCEPTION model on the FER2013.

Emotional Category	Sample Number	Precision/%	Recall/%	F1/%
Anger	491	64.18	63.14	63.66
Disgust	55	38.60	40.00	39.29
Fear	528	59.91	52.08	55.72
Sadness	879	89.18	85.32	87.21
Surprise	594	59.32	58.92	59.12
Happy	416	69.62	79.33	74.16
Neutral	626	64.23	70.29	67.12

**Table 4:** Experimental results comparing the performance of the Mini_XCEPTION model before and after INT8 quantization.

Model Version	Data Type	Model Size	Compression Ratio	Inference Time	Accuracy Change
Mini_XCEPTION (Origin)	FP32	246 KB	1×	32 ms (~31 FPS)	Reference
Mini_XCEPTION (Quant)	INT8	68 KB	3.6×	21 ms (~47 FPS)	−0.8%

**Table 5:** Ablation comparison of class weighting and robust loss functions on the FER2013 validation set.

Loss Function	Class Weighting	Gaussian Smoothing	Accuracy	Macro-F1	Anger Recall	Fear Recall	Disgust Recall
CE	No	No	68.2%	58.1%	51%	45%	42%
WCE	Yes	No	72.1%	63.8%	62%	55%	58%
Focal Loss	No	No	70.6%	61.3%	57%	51%	52%
Class-Balanced Focal Loss	Yes	No	72.5%	64.7%	64%	57%	60%
GSWCE	Yes	Yes	73.2%	66.1%	66%	59%	62%

**Table 6:** Cross-domain test results of the models.

Model Configuration/Test Set	Overall Accuracy	Mean Recall of Majority Classes (Happy/Neutral)	Mean Recall of Negative Classes (Anger/Fear/Sad)
Baseline Model (Standard CE, No Preprocessing) FER2013	68.2%	84.5%	46.8%
Improved Model (GSWCE + Preprocessing) FER2013	73.2%	81.2%	61.5%
Improved Model (GSWCE + Preprocessing) Real In-Cabin Test Set (Zero-shot)	62.8%	73.4%	54.2%

**Table 7:** Impact analysis of sliding window parameters on system latency and false trigger rate.

Window Size (N)	Voting Threshold (θ)	Average Latency (ms)	False Trigger/ Jitter Rate (%)	Comprehensive Evaluation
1	-	~21	28.5%	Extremely low latency, highly unstable system
3	0.6	~150	14.2%	Low latency, occasional jitters remain
5 (Proposed)	0.6	~250–300	4.1%	Acceptable latency, high stability
10	0.6	~500–600	1.8%	Extremely stable, excessive latency causes lagging

**Table 8:** Matrix of mapping strategies for driver emotions and interior ambient lighting effects.

Emotion Category	Valence–Arousal Features	Estimated Associated Driving State	Target Lighting Atmosphere	Colour and Initial HSV Parameters	Psychological/Environmental Ergonomics Design Hypothesis
Anger	Negative valence/high arousal	High probability association with aggressive/road rage behaviour	Calm/ Relaxing	Cyan (180°, 0.4, 0.6)	Low-saturation cool tones act as a heuristic visual buffer. This provides calmness and avoids secondary stimulation from high-saturation warm colours.
Disgust	Negative valence/high arousal	Associated with driving dissatisfaction or cognitive conflict	Soothing/ Harmonizing	Lavender (270°, 0.5, 0.6)	Lavender blends calming cool and integrating warm tones. This transitional light aims to subconsciously soften cognitive conflict and driver dissatisfaction.
Fear	Negative valence/very high arousal	Associated with acute stress or panic response	Safe/ Comforting	Amber (40°, 0.6, 0.5)	Amber simulates the warm 2000 K colour temperature of candlelight. This creates a spatial sense of safety to help alleviate acute driving anxiety.
Sadness	Negative valence/low arousal	Preliminary proxy indicator for low arousal states (fatigue/ mind wandering)	Arousing/ Alerting	Sunset Orange (30°, 0.8, 0.7)	Sadness serves as a heuristic proxy for low-arousal states like fatigue. High-brightness warm tones act as visual stimuli to counteract cognitive disengagement.
Surprise	Ambivalent valence/high arousal	Associated with unexpected road conditions or attention interruption	Focusing/ Centring	Cold White (0°, 0.0, 0.9)	High-intensity, 5000 K+ cold white light leverages non-visual biological effects (when driver is tired). This briefly elevates visual attention to help assess unexpected road conditions rapidly.
Happy	Positive valence/ moderate arousal	Normal or pleasant driving state	Sustaining/Rhythmic	Dynamic Rhythm	Based on the Yerkes–Dodson Law, dynamic rhythms maintain optimal arousal. This heuristically enhances the subjective and pleasant driving experience.
Neutral	Neutral valence/ moderate arousal	Baseline driving state	Comfortable/Natural	Champagne (45°, 0.3, 0.5)	Low-saturation warm white light serves as the ergonomic baseline state. This natural visual environment aims to reduce prolonged driving fatigue.

**Table 9:** Recovery-time comparison under control and lighting-intervention conditions (N = 12).

Scenario	Control Group	Experimental Group	T (11)	*p*-Value	95% CI of Difference	Cohen’s d
Anger Recovery Time	17.1 ± 2.8 s	9.8 ± 1.5 s	8.65	<0.001	[5.4, 9.2]	3.2
Arousal Recovery Time	27.5 ± 4.3 s	12.5 ± 2.1 s	11.20	<0.001	[12.0, 18.0]	4.3

## Data Availability

The original contributions presented in this study are included in the article. Further inquiries can be directed to the corresponding author.
