# Correction: He et al. An Edge-Computing-Based Emotion-Aware Adaptive Lighting System for Intelligent Cockpits. *Sensors* 2026, *26*, 3489

**DOI:** 10.3390/s26134025

**Published:** 2026-06-25

**Authors:** Lei He, Ning Jia, Jiaqi Zhao

**Affiliations:** National Key Laboratory of Automotive Chassis Integration and Bionics, Jilin University, Changchun 130025, China; jianing24@mails.jlu.edu.cn (N.J.); jiaqiz23@mails.jlu.edu.cn (J.Z.)

## Institutional Review Board Statement Correction

There was an omission in the original publication [1]. The Institutional Review Board Statement was not included in the manuscript before acceptance. A correction has been made to add the Institutional Review Board Statement in the back matter of the article, in the section “Institutional Review Board Statement.”

The reason for this correction is that the Institutional Review Board Statement was unintentionally omitted from the final accepted manuscript. The study involved human participants in driving simulation and emotion-related experiments, and the ethical review and approval had been obtained before the experiments were conducted. All participants were informed of the research purpose, experimental procedure, data usage, privacy protection measures, and their right to withdraw from the study at any time without penalty.

The authors state that the scientific conclusions are unaffected. This correction was approved by the Academic Editor. The original publication has also been updated.

The correct statement appears below.

Institutional Review Board Statement: The study was conducted in accordance with the Declaration of Helsinki and approved by the Institutional Review Board of College of Automotive Engineering, Jilin University (No separate protocol code was issued for this study, and the date of approval was 11 February 2026).
